# Case Report: Novel treatment approach for severe interstitial lung disease in type 3 Gaucher disease

**DOI:** 10.3389/fped.2025.1604433

**Published:** 2025-06-11

**Authors:** Vincenza Gragnaniello, Silvia Carraro, Tiziana Zangardi, Chiara Cazzorla, Daniela Gueraldi, Alberto B. Burlina

**Affiliations:** ^1^ Division of Inherited Metabolic Diseases, Department of Women's and Children's Health, University of Padova, Padova, Italy; ^2^ Division of Inherited Metabolic Diseases, Department of Women's and Children's Health, University Hospital of Padova, Padova, Italy; ^3^ Unit of Pediatric Allergy and Respiratory Medicine, Women’s and Children’s Health Department, University of Padova, Padova, Italy; ^4^ Pediatric Emergency Department, Department for Women’s and Children’s Health, University of Padua, Padua, Italy

**Keywords:** Gaucher disease, glucocerebrosidase, newborn screening, interstitial lung disease, corticosteroid therapy, hydroxychloroquine, inflammation, enzyme replacement therapy

## Abstract

Gaucher Disease Type 3 (GD3) is a rare lysosomal storage disorder characterized by both visceral and neurological involvement. Pulmonary manifestations can significantly impact prognosis and quality of life. This case report highlights the challenges in managing severe pulmonary involvement in GD and explores novel treatment approaches. We present a case of a patient with GD3, diagnosed through neonatal screening, who developed severe lung disease despite early initiation of enzyme replacement therapy (ERT). The patient, carrying compound heterozygous variants in the *GBA1* gene (p.Leu483Pro, [p.His294Gln + p.Asp448His]), experienced respiratory distress requiring oxygen therapy from the age of 4 months. High-resolution computed tomography revealed a typical interstitial lung disease pattern. Despite ERT and a marked reduction in storage biomarkers, pulmonary symptoms persisted, accompanied by elevated inflammatory markers. We implemented a treatment regimen of systemic corticosteroids followed by hydroxychloroquine, resulting in clinical improvement. Furthermore, we observed a decrease in inflammatory biomarkers, such as TNF-alpha and Pp38 MAPK levels, providing insights into possible pathogenic mechanisms. This case underscores the limitations of ERT in addressing pulmonary manifestations of GD and highlights the need for personalized treatment strategies. It also emphasizes the importance of further research into the pathogenesis of pulmonary damage in Gaucher disease to develop more effective therapies for these challenging cases. The positive response to anti-inflammatory and immunomodulatory therapies suggests a potential role for these approaches in managing GD-related lung disease.

## 1 Introduction

Gaucher disease (GD) is an autosomal recessive disorder caused by mutations in the *GBA1* gene, which encodes the lysosomal enzyme glucocerebrosidase (GCase). GCase catalyzes the hydrolysis of glucosylceramide (GluCer) to ceramide and glucose. Its deficiency results in the accumulation of GluCer within lysosomes, especially in macrophages (Gaucher cells) (1–3).

GD presents with a wide phenotypic spectrum, ranging from non-neuronopathic forms (GD1) to acute (GD2) or chronic (GD3) neuronopathic forms. GD3 can manifest with visceral involvement characterized by hepatosplenomegaly, cytopenia, bone marrow infiltration, and skeletal involvement, as well as slowly progressive neurological deterioration of variable onset and severity. Neurological manifestations may include supranuclear horizontal ophthalmoplegia, progressive myoclonic epilepsy, cerebellar ataxia, spasticity, and developmental delay (4–6).

Enzyme replacement therapy (ERT) is effective in treating non-neurological manifestations of GD3. While some evidence suggests initiating therapy for symptomatic patients (7), other studies indicate that specific treatment with ERT should be considered for all GD3 patients (8, 9). This recommendation extends to presymptomatic siblings of GD3 patients, particularly those with specific genotypes such as homozygous or compound heterozygous p.Leu483Pro and p.Asp448His variants (10).

Pulmonary involvement in GD can range from clinically asymptomatic with normal or mild radiographic changes to severe respiratory symptoms with significant radiographic findings (11). Infiltrative lung involvement, including interstitial lung diseases and rapidly progressive lung consolidation, is caused by Gaucher cells infiltrating the perivascular, peribronchial, septal regions, and alveolar air spaces.

Chest X-rays may show diffuse interstitial and nodular infiltrates, with honeycombing. However, the diagnostic gold standard is high-resolution computed tomography (HRCT), which typically reveals ground-glass opacities, sometimes superimposed with interlobular and intralobular septal thickening.

Despite the availability of enzyme replacement therapy (ERT), the course of the lung disease is associated with high morbidity and mortality. Patients frequently require long-term oxygen therapy and sometimes artificial ventilation (12).

In these cases, a deeper understanding of the pathogenesis of the damage and the use of personalized therapies is necessary. We describe the case of a patient with GD3 identified through neonatal screening, who suffered from severe interstitial lung disease poorly responsive to early ERT. The patient was successfully treated with anti-inflammatory and immunomodulatory therapy.

## 2 Case description

The patient is the third child born to non-consanguineous parents; the father is of Balkan origin, and the mother is Italian. At birth, extended neonatal screening, active in Northeast Italy for lysosomal diseases since 2015 (13, 14), showed reduced GCase activity (0.84 µmol/L/h, normal value >2.5) and elevated Lyso-Gb1 values (1,436 nmol/L, normal value 5.64–33.31) on dried blood spot (DBS), suggesting GD.

The diagnosis was confirmed by reduced GCase activity in lymphocytes (0.08 µmol/h/mg protein, normal value 0–1.93 µmol/h/mg protein) and elevated Lyso-Gb1 levels in plasma (182 µmol/L, normal value <1.93 µmol/L). Molecular testing of the *GBA1* gene, become available when the child was 2 months old, revealed compound heterozygosity for the allele c.882T > G + c.1342G > C (p.His294Gln + p.Asp448His) and the allele c.1448T > C (p.Leu483Pro). Both alleles have been associated with acute and chronic neurovisceral phenotypes of Gaucher disease (15, 16) (for methods, see Supplementary Materials).

### 2.1 Clinical course

At the first assessment, the newborn showed no signs or symptoms of the disease, specifically no cytopenia (Hb 13.3 g/dl, PLT 207,000/mm^3^) and no visceromegaly on abdominal ultrasound. He presented no neurological signs or ocular abnormalities, and EEG, ABR, and brain MRI were unremarkable.

**Figure 1:**
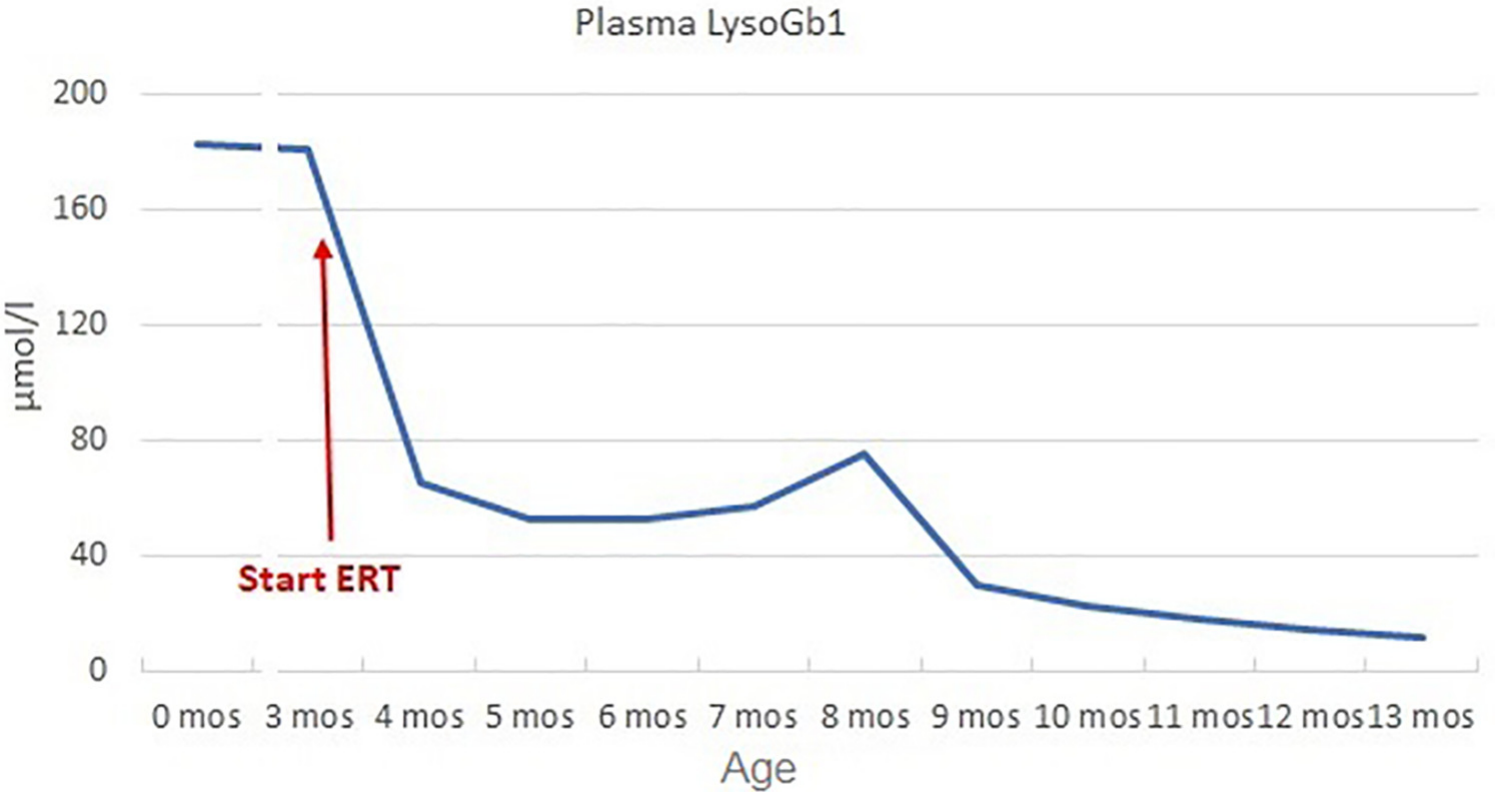
Plasma LysoGb1 trend. LysoGb1 levels rapidly decreased after the initiation of ERT (from 180.7 to 65.4 umol/L, −64% after 1 month), then slowly reduced until reaching 13.9 umol/L at 13 months of age (+10 months of ERT).

At 3 months, the patient had persistently elevated biomarkers (plasma Lyso-Gb1 180.7 µmol/L), mild splenomegaly on abdominal ultrasound (longitudinal diameter 6 cm, >90 pc), and mild axial hypotonia with partial head control. Hematological parameters remained normal (Hb 10.2 g/dl, PLT 358,000/mm^3^). Considering the disease signs and the genetic results suggestive of a severe form (8–10), ERT was initiated at 60 U/kg every other week, along with ambroxol 25 mg/kg in five doses/day as chaperon therapy (17). Biochemical response was good (reduction of Lyso-Gb1 of 64% after 1 month, Figure 1) and spleen volume remained unchanged. Psychomotor development progressed normally (Bayley III: cognitive 100, language 97, motor 88) and no neurological symptoms appeared.

### 2.2 Pulmonary manifestations

During the second month of ERT, the patient presented with dyspnea, tachypnoea (RR 50–60 breaths per minute), and oxygen desaturation. Pulse oximetry monitoring revealed a lowest SatO2 of 85% in the supine position. Chest auscultation appeared normal. Chest x-ray was normal, and nasopharyngeal aspirate was negative for main respiratory viruses (adenovirus, bocavirus, enterovirus, metapneumovirus, parechovirus, parainfluenza, rhinovirus, SARS-CoV-2, RSV). Cardiological evaluation, including ECG and echocardiography, ruled out a cardiogenic cause of desaturation.

**Figure 2:**
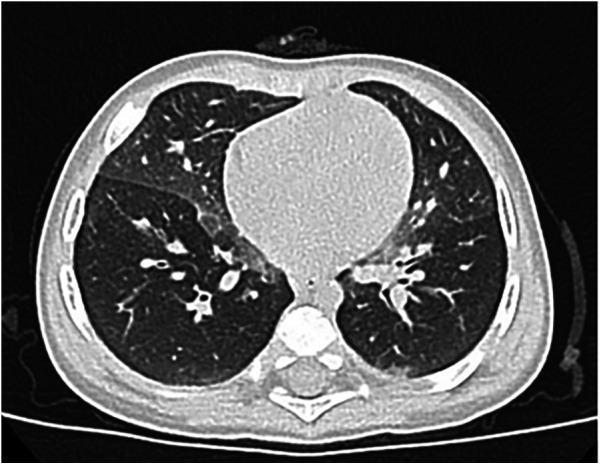
Chest HRCT: heterogeneous pulmonary density with a “mosaic” pattern, characterized by the presence of confluent “ground-glass” areas, suggestive of alveolar interstitial disease.

In the following days, the patient suffered from tachypnoea and dyspnea. The patient was continuously monitored with a pulse oximeter and intermittent oxygen therapy was initiated when saturation consistently fell below 92%. These desaturation episodes were more frequent when the patient was in a supine position and were not associated with feeding. Although the patient did not have increased respiratory secretion, ambroxol therapy was discontinued for a week with no changes, so it was reintroduced. EEG showed no electrical correlates. Polysomnography showed tonic desaturation with a mean pulse oximetry SatO_2_ of 90%. Nocturnal transcutaneous pCO2 monitoring did not show hypercapnia (median pCO2 46 mmHg, range 42–49). Chest HRCT revealed heterogeneous pulmonary density with a “mosaic” pattern, characterized by confluent “ground-glass” areas, predominantly distributed in the perihilar and subpleural regions. Furthermore, the peribronchal interstitium was mildly thickened along the perihilar segmental branches, particularly in the right upper lobe. These findings were suggestive of alveolar interstitial disease typical of GD (Figure 2). The ERT dose was increased to 90 U/kg/week based on isolated reports suggesting improved efficacy with high-dose ERT (18).

However, the severe pulmonary involvement [ILD staging 4 according to European protocols (19)] was poorly responsive to high-dose ERT. We hypothesized that the damage could be amplified by the inflammatory state induced by accumulation in Gaucher cells. Indeed, despite ERT, the patient exhibited elevated markers of systemic inflammation (TNF-alpha 28.5 ng/L, normal value < 8.1) and cellular stress. Specifically, the level of Pp38 MAPK in peripheral blood mononuclear cells (PBMCs) (20) was considerable (4.8 times higher than control).

**Figure 3:**
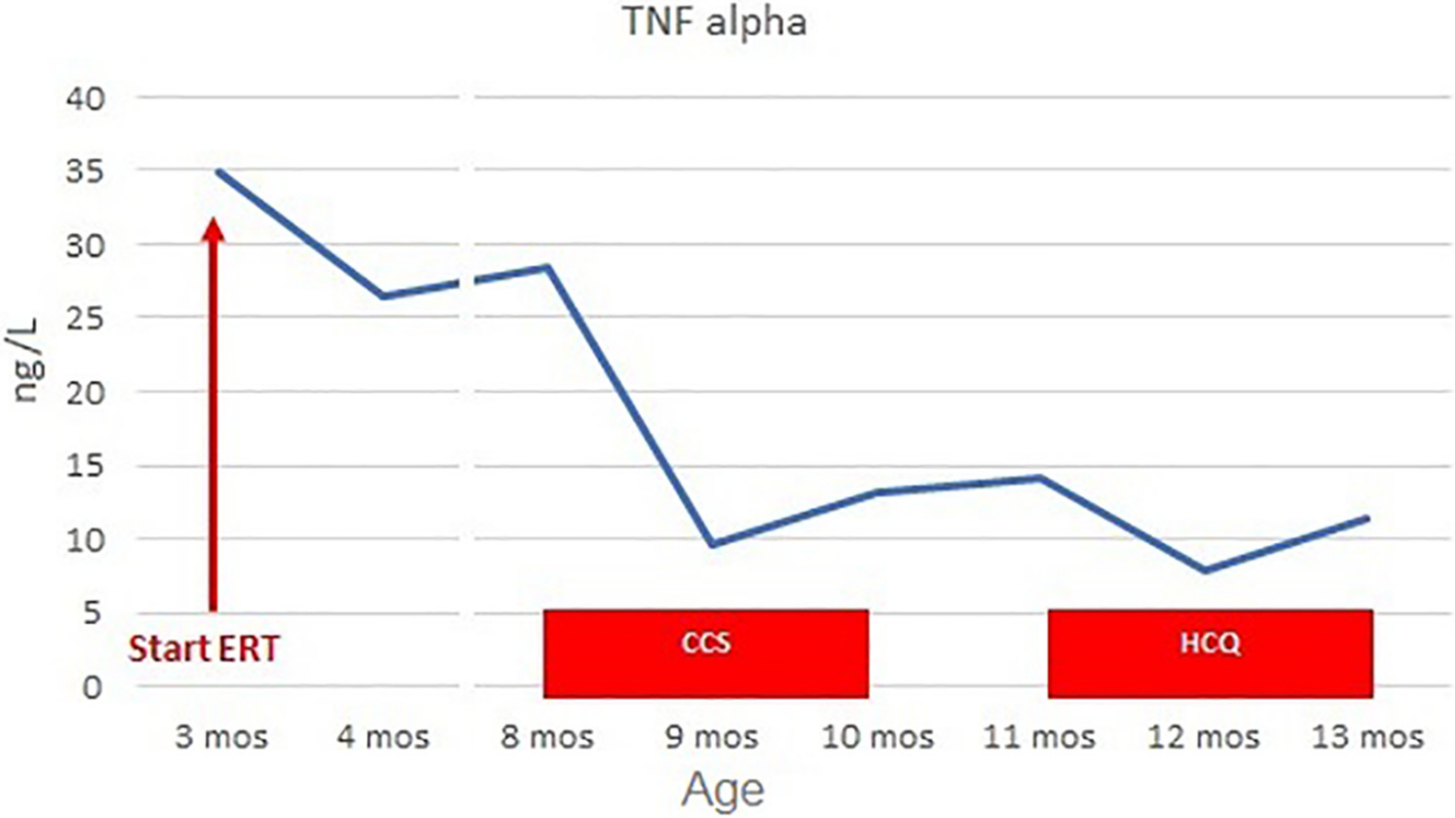
TNF-alpha levels before and during CCS/HCQ therapy. TNF-alpha levels decreased from 28.5 ng/L (normal value <8.1) to 9.6 ng/L during CCS therapy. They slightly increased to 14.1 ng/L at the end of therapy, then decreased again during HCQ treatment to 7.8 ng/L after the first month and 11.3 ng/L after the second month.

**Figure 4:**
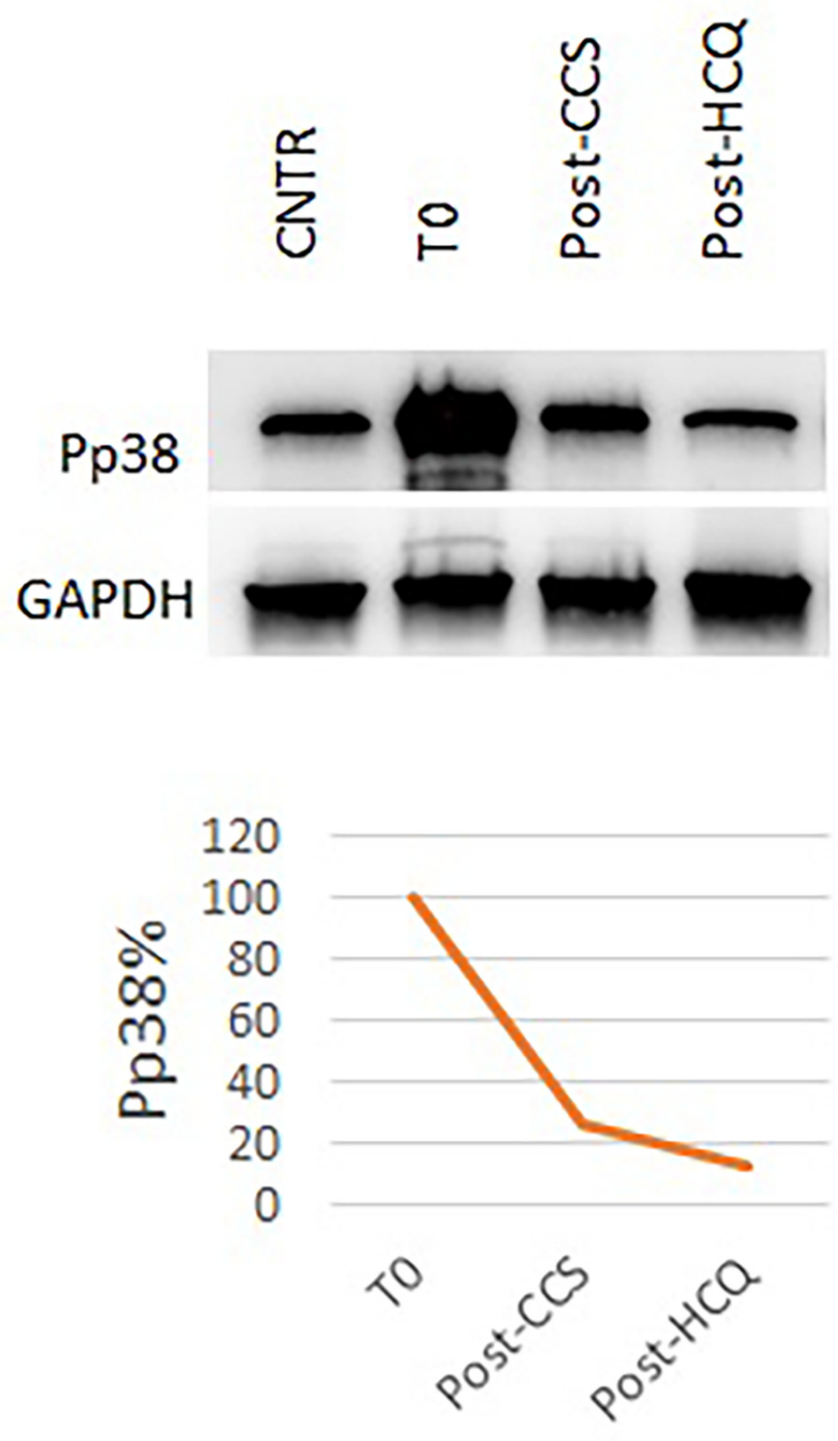
Pp38 levels before and during CCS/HCQ therapy. P-p38 levels decreased by 72% after CCS treatment and underwent a further slight reduction (−88% compared to baseline) after HCQ treatment, reaching values equal to the healthy control.

For these reasons, at the age of 8 months, we initiated steroid therapy with oral methylprednisolone 2 mg/kg/day, tapered by 0.25 mg/kg/week and stopped after 8 weeks, at the age of 10 months (21). The patient experienced clinical improvement, with improvements in home pulse oximetry readings and reduced oxygen requirements at home (ILD staging 3). Concurrently, TNF-alpha levels decreased (9.6 ng/L at 1 month, 13.2 ng/L at 2 months, Figure 3) and Pp38 MAPK levels in PBMCs decreased by 72% at the end of the cycle (2 months) (Figure 4).

However, clinical benefits began to diminish as we tapered the steroid dose. Polysomnography at the end of steroid tapering showed no significant improvements from baseline (mean pulse oximetry SatO2 of 88.6%). Due to the transient benefit from steroid therapy, at 11 months of life, the patient started hydroxychloroquine 6 mg/kg/day, in accordance with European protocols for managing interstitial lung disease in children. Before starting therapy, ECG and ophthalmological examination were normal; chest x-ray showed a slight accentuation of the interstitial pattern in the lower lung.

After 2 months (age of 13 months), the patient showed clinical improvement (ILD staging 1). Chest x-ray showed only a slight accentuation of the perihilar texture and polysomnography was normal (mean pulse oximetry SatO2 of 98%). TNF-alpha levels slightly decreased (T0 14.1 ng/L, T1 month 7.8 ng/L, T2 months 11.3 ng/L, Figure 3). P-p38 levels in PBMCs decreased by 88% compared to the baseline values at the start of steroid therapy, reaching levels comparable to those of the control (Figure 4). Given the observed functional improvements, we chose not to repeat CT scans in the short term due to concerns about cumulative radiation exposure in a pediatric patient.

The only adverse event observed was an increase in urea (from 3.20 mmol/L to 7.70–7.90 mmol/L, normal value 1.8–6.4), with consistently normal creatinine levels (20–21 µmol/L, normal value 15–31).

## 3 Discussion

We describe the case of a patient with GD3, diagnosed through neonatal screening and early treated with ERT and chaperone therapy. Despite this, the patient developed severe lung disease with respiratory distress, requiring oxygen therapy.

Pulmonary involvement is more frequent in GD2 and GD3 patients (1, 22, 23), especially in those carrying the p.Leu483Pro variant, as our patient. Consistent with our case, primary lung disease is likely to occur at early ages, even in subjects with no overt neurological disease. It is related to a higher disease severity (11, 22, 24).

While improvements in pulmonary function tests and some clinical parameters have been reported in individuals receiving high-dose ERT, the general experience is that pulmonary manifestations are slow to respond to enzyme treatment, and some damage may not be reversed (12, 25, 26). Several cases of neuronopathic GD with progressive respiratory symptoms despite ERT have been described, necessitating tracheotomy and artificial ventilation (27). Few cases of lung transplantation have also been reported (26).

The inadequate response to ERT is likely attributable to poor accessibility of the infused enzyme to the lungs, as confirmed by post-mortem examinations. Burrow et al. reported on a 12.5-year-old GD type 3 patient who had received 11 years of ERT. The autopsy revealed clusters of macrophages and very elevated glucosylceramide (GluCer) and glucosylsphingosine (GluS) levels in the lungs and lymph nodes, while the liver and spleen were clear of storage cells and had nearly normal GluCer and GluS levels. The pathological features of the lungs were essentially identical to those of untreated GD3 patients, suggesting that ERT may not effectively penetrate (28). This highlights the need for additional therapeutic approaches and strategies based on a better understanding of lung damage pathogenesis.

It is known that in GD, chronic constitutive macrophage activation may lead to persistent immune system stimulation and systemic inflammation (29, 30). In a previous study, we demonstrated that TNF-alpha levels and P-p38 are sensitive markers of inflammation and cellular stress in Gaucher disease, showing alterations even in the presymptomatic phase (20). Other Authors have reported elevated plasma levels of TNF-alpha and other cytokines in GD patients, with highest concentrations in those with neuronopathic forms (31). Our patient also showed increased TNF-alpha levels without concomitant infections.

TNF-alpha is a pleiotropic cytokine that regulates several pathways, including the MAPK pathway (32). We investigated the activation of the proinflammatory kinase p38 in our patient, which was strongly elevated compared to the control before steroid therapy. Previous *in vivo* studies have demonstrated p38 activation in GD mice, showing increased P-p38 levels in brain tissues of neuronopathic mice and other affected organs such as liver and lung across all types of GD (33). Overactive p38 contributes to the generation of various inflammatory mediators, amplifying the inflammatory milieu (34).

The effect of steroids in other inflammatory lung diseases has been already reported, including their effects on cytokine production and MAPK pathways. It has been demonstrated that the extent of TNF-induced p38 MAPK phosphorylation is reduced in cells treated with dexamethasone. Indeed, the MAPK deactivator—mitogen-activated protein kinase phosphatase 1 (MKP-1)—is a corticosteroid-inducible gene (35–37). Consistently, our patient showed a reduction in TNF-alpha levels and P38 activation after steroid therapy. In particular, TNF-alpha levels were notably reduced after the first month of therapy, coinciding with clinical improvement. However, they subsequently increased during the tapering phase, concurrent with a return to baseline clinical conditions.

Due to the transient clinical benefit with corticosteroids, we used hydroxychloroquine for long-term treatment. Indeed, it is generally well-tolerated with relatively few side effects.

Interestingly, hydroxychloroquine appears to have an unfavorable effect on lysosomal function, inhibiting lysosomal acidification and preventing autophagosome degradation (38). Nevertheless, it proved effective in our patient. This observation, coupled with the further improvement in inflammation and cellular stress markers, leads us to hypothesize that the mechanism of action is primarily related to the immunomodulatory effect of the drug. The exact mechanism of action for its immunomodulatory effect is not fully understood, but it seems to include decreased macrophage-mediated cytokine production, especially IL-1 and IL-6 (19, 39).

Our patient showed an optimal clinical response after 2 months of therapy, with normal ventilatory pattern on polysomnography and further improvement in inflammatory biomarkers.

In conclusion, pulmonary involvement is crucial in determining the prognosis in neuronopathic Gaucher disease and can greatly influence the patient's quality of life. Although controlling respiratory symptoms is important, it is extremely challenging to achieve with currently available therapies. Our case underscores the potential role of inflammation in the pathogenesis of lung damage and the possible benefits of targeting the inflammatory pathways involved. The positive response to steroid therapy and hydroxychloroquine opens avenues for further research into these treatment modalities.

## Data Availability

The original contributions presented in the study are included in the article/Supplementary Material, further inquiries can be directed to the corresponding author.
