# Supplementary material for: Case Report: Novel treatment approach for severe interstitial lung disease in type 3 Gaucher disease
Source: Front Pediatr. 2025 Jun 11;13:1604433. doi: 10.3389/fped.2025.1604433 (PMC12187823; doi:10.3389/fped.2025.1604433)
Supplement: Supplementary file 1 [file Table1.docx]

**Supplementary Materials:**

**Materials and Methods:**

Biochemical Tests:

GCase activity was measured in dried blood spots and lymphocytes using the NeoLSD kit from Perkin Elmer (Turku, Finland) and MS/MS, as previously reported [Burlina 2018]. LysoGb1 levels in dried blood spots and plasma were assayed by LC-MS/MS, following previous reports [Polo 2017, Polo 2019]. TNFα was assayed using the human TNFα ELISA kit (Invitrogen), following the manufacturer's instructions.

Molecular Testing:

Genomic DNA was extracted from leukocytes, and next-generation sequencing (NGS) was performed using the Illumina MiSeq Sequencing System to analyze specific exon regions as well as exon-intron boundaries. Identified variants were annotated against the human *GBA1* reference sequence (NM_000157.4). We followed the standard naming convention of the Human Genome Variation Society for the GBA1 gene, designating methionine encoded by the translation initiation codon as position 1 in amino acid numbering, in contrast to the common GBA1 variant naming which omits the GBA1 signal peptide residues.

Immunoblotting:

The cell pellet obtained after centrifugation was used to isolate peripheral blood mononuclear cells (PBMCs) by density gradient centrifugation over Ficoll (Cytiva). PBMCs were analyzed by immunoblotting to determine the levels of P-p38 (the activated form of p38 MAPK).PBMC extracts were resuspended in RIPA buffer (phosphate-buffered saline PBS with 1% NP-40, 0.5% sodium deoxycholate, 0.1% sodium dodecyl sulfate SDS) supplemented with 1% protease inhibitors (Thermo Fisher), disrupted by vortexing and centrifuged at 1300 rpm for 30 min at 4 °C. Protein concentration was measured by BCA (bicinchoninic acid) assay according to manufacturer’s instructions (PierceTM BCA Protein Assay kit, Thermo Fisher). Equal amounts (20 ug of protein) of extracts were heat-denatured for 5 min at 95 °C and subjected to sodium dodecyl sulfate polyacrylamide gel electrophoresis (4–15% polyacrylamide). PBMC extracts from healthy subjects were run in parallel for comparison. Proteins were transferred to a nitrocellulose membrane, that was incubated with blocking solution (Everyblot, Bio-Rad) for 5 min. Then, it was incubated with the primary antibody (anti-p-p38 rabbit polyclonal antibody) overnight at 4 °C and subsequently with secondary antibodies (horseradish peroxidase HRP conjugated antirabbit IgG) for 1 h. Immunoreactive proteins were detected by chemiluminescence (ECL, Bio-Rad). The GAPDH peptide allowed comparison of different samples. All reagents (blocking solution, precast gels, nitrocellulose membranes, TBS-Tween 20, ECL) and instruments (Transblot, Chemidoc) were from Bio-Rad. Primary antibodies were from Cell Signaling (p-p38) or Biorad (GAPDH) and used with a dilution of 1:1000. Secondary antibodies were from Biorad and used with a dilution of 1:2000. Quantitative analysis of band intensity was performed using Image Lab.

Burlina AB, Polo G, Salviati L, et al. Newborn screening for lysosomal storage disorders by tandem mass spectrometry in North East Italy. J Inherit Metab Dis. 2018 Mar;41(2):209-219.

Polo G, Burlina AP, Kolamunnage TB, et al. Diagnosis of sphingolipidoses: a new simultaneous measurement of lysosphingolipids by LC-MS/MS. Clin Chem Lab Med. 2017 Mar 1;55(3):403-414.

Polo G, Burlina AP, Ranieri E, et al. Plasma and dried blood spot lysosphingolipids for the diagnosis of different sphingolipidoses: a comparative study. Clin Chem Lab Med. 2019 Nov 26;57(12):1863-1874.
